# Marine abundance and its prehistoric past in the Baltic

**DOI:** 10.1038/s41467-022-30150-9

**Published:** 2022-05-20

**Authors:** Niklas Hausmann, Harry K. Robson, Geoff Bailey

**Affiliations:** 1 Römisch Germanisches Zentralmuseum (RGZM) Leibniz Research Institute for Archaeology, Mainz, Germany; 2 Department of Archaeology, University of York, King’s Manor, York, YO1 7EP UK; 3 College of Humanities, Arts and Social Sciences, Flinders University, Adelaide, Australia

**Keywords:** Climate-change adaptation, Environmental impact

**arising from** Lewis et al. *Nature Communications* 10.1038/s41467-020-15621-1 (2020)

In a recent article, Lewis et al.^1^ advance the hypothesis that an increase in the marine fertility of Danish waters from ca. 7600 cal BP onwards fuelled an intensification in the marine economy and a fourfold population increase in the later Mesolithic period. This hypothesis is severely compromised by: (a) reliance on archaeological data from shell middens without reference to the multiple biases that operate differentially to distort quantitative inferences from such deposits, (b) selective use of stable isotope data obtained from human bone collagen and dates concerning marine technology, and (c) the assumption that human economic choices closely or necessarily track environmental change.

We conclude that these biases cast doubt on the case for Late Mesolithic intensification and population increase, and that investigation of the undoubtedly complex interactions between environmental change and human response requires wider multi-disciplinary collaboration, better integration and understanding of palaeoecological, archaeological, geoscientific and biomolecular datasets, better recognition of their limitations, greater attention to the differential taphonomic histories of archaeological sites and materials, and better articulation and evaluation of alternative hypotheses.

## Critical points

### Shell accumulations and radiocarbon dates as proxy measures of marine consumption

Lewis et al. (their Fig. 2e) present a measure of shell accumulation based on radiocarbon dates from oyster shells found in Danish shell middens ‘as a proxy for human coastal marine utilisation, and by implication, marine resource availability’ (p. 3).

Shell middens are highly variable in size and volume, ranging from small shell scatters to mounds with thousands of cubic metres of shell^2^. As such, radiocarbon dates represent shell layers of varying volume and quantity of shells^3^. Quantification of the relationship between numbers of ^14^C dates and sizes of shell deposits shows great variability. In Denmark, the Visborg shell midden covers ca. 18,000 m^2^, with only 16 shell-derived ^14^C dates published so far^4–6^, a ratio of ^14^C dates to square metres of shell deposit of roughly 1:1000. At the Ertebølle shell midden, which was dated using 34 ^14^C dates and which has a size of ca. 2800 m^2^, the ratio is ca. 1:100, an order of magnitude smaller^7,8^. For other well-known shell middens in Denmark the ratio is ca. 1:11 at Norsminde (ca. 360 m^2^, 32 dates)^9^ and ca. 1:355 at Bjørnsholm (ca. 11,000 m^2^, 31 dates)^10^. Reliance on radiocarbon dates to quantify shellfish remains thus only works in the rare case that sites are similar with respect to the proportion of the deposits excavated and sampled for dates, as well as taphonomic histories of visibility and preservation, or where these variables are known and can be controlled for. Additional variables are site function, distance from the source of the shell food^11^, and post-depositional loss of shell^12–14^. Post-depositional loss of shell has demonstrably occurred at the Danish shell middens of Bjørnsholm^10^, Brovst^15^, Ertebølle^8^, Hjarnø^16^, Krabbesholm II^17^ and Visborg^5,6^ either because of marine erosion or anthropogenic destruction in the form of ploughing.

This necessary information about potentially large sample biases is not presented in the study by Lewis et al., making it impossible to evaluate the relationship between the radiocarbon dates and relative changes in marine consumption or resource availability.

Moreover, Lewis et al.^1^ state that there is an ‘absence of any other reliable method of quantifying shell midden abundance or volume’ (p. 5, Supplementary Information). This statement is incorrect given the work by Stein et al.^3^ and other archaeological studies referenced above.

### Sea-level variation and site preservation

In relation to the shell accumulation curve as well as further points below, it is important to consider the role of sea-level change in site preservation, which is missing from the study.

**Fig. 1:**
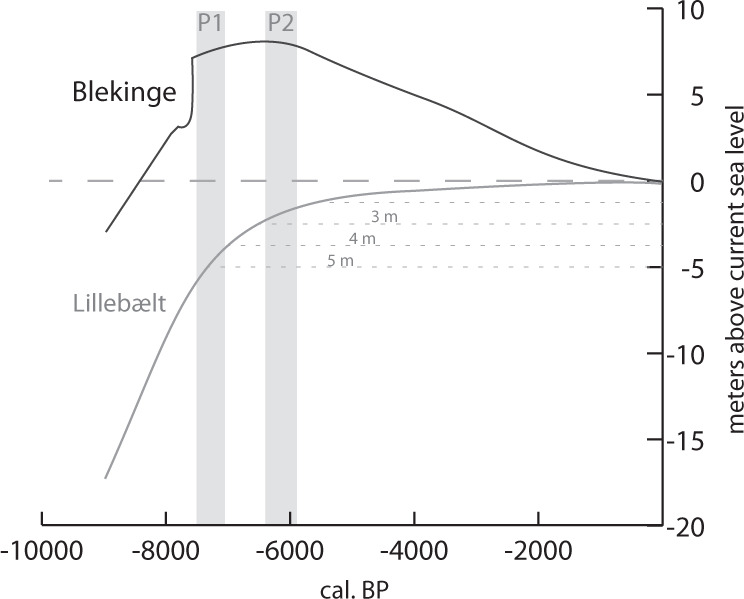
Sea-level curves from the Baltic Sea. The curve used by Lewis et al. (Blekinge, Sweden) and the more southern curve (Lillebælt, Denmark) over the last 9000 years. P1 and P2 indicate times of increased marine production as suggested by Lewis et al. (data from Rosentau et al.^39^ and references therein).

Shell middens located on the immediate shoreline are vulnerable to destruction by coastal erosion especially during relative sea-level rise, resulting in geographical and temporal gaps in site distribution^11^. The sea-level curve used by Lewis et al.^1^ is from Blekinge in southern Sweden, a region of glacio-isostatic uplift. While this curve is ‘broadly representative’ (p. 13, Supplementary Material) for northern Denmark, where many of the known shell middens are concentrated, there is a progressive transition from uplift in the north to submergence in the south, resulting in different sea-level curves further south (Fig. 1). In the southern half of the country, all Mesolithic shorelines are submerged^18^. Rare underwater shell middens are known, notably at Hjarnø, one of the earliest dated shell middens in the Danish sequence at ca. 7400 cal BP, where the shell midden layer has been truncated, with removal of some midden shell, and redeposition and mixing with marine sediment, reducing the surviving midden to a fraction of its original size^19^. We do not know how many more existed on the now submerged palaeoshorelines and were subsequently damaged or destroyed by erosion or buried under marine sediment. However, given the large number of known submerged finds and non-shell-bearing sites^20^, it is certain that there is a substantial gap that overlaps with the study period and some of the study region.

Moreover, the current submergence is of only secondary importance. All shell middens which pre-date the time of the high sea-level stand at ca. 6500 cal BP would have been especially vulnerable to marine erosion — including sites in the uplifted north, despite the fact that their locations are now above modern sea level. Some early sites in the north such as Brovst (ca. 7600 cal BP) have survived but with demonstrable evidence of exposure to marine erosion^15^. Therefore the rarity of early sites, whether in submerged or uplifted locations, is linked to marine erosion, which is sufficient to account for the marked increase of preserved oyster dates after ca. 6400 cal BP (P2 in Fig. 1, and Fig. 2c and 2e of Lewis et al.). There is, therefore, a systematic bias against the representation of shell middens in the earlier part of the Mesolithic sequence that needs to be recognised.

### Oyster shells as a proxy for resource availability and the wider marine economy

Lewis et al. state that, ‘[Intense marine resource exploitation] is shown here by the widespread development of large, accessible shell beds in Danish inner waters (Fig. 2e) and, by implication, other marine resources such as mammals, fish and birds’ (p. 7). This sentence involves two unwarranted assumptions.

The first assumption is that shell midden data for quantities of consumed oysters are a reliable proxy for quantities of available oysters. Without independent evidence for the quantities available, this is a circular argument. Economic choices are driven by many different socio-economic and cultural pressures, resulting in avoidance or exploitation of a particular resource regardless of its availability^21,22^.

The second assumption is that oysters are a reliable proxy for other marine resources. The major difficulty here is that oysters made a relatively small contribution to coastal palaeodiets^23,24^. The consensus is that fish, not shellfish, are the dominant marine resource in the Mesolithic coastal economy and the principal determinant of population size^25,26^. As the authors state, there was a wide range of marine resources available to the Mesolithic communities. However, no comparison in the change of the local fish fauna and oyster quantities during the Mesolithic is carried out, without which we can see no evidence for assuming that the latter can be used as a proxy for the former. Moreover, according to Andersen^2^, shell middens are outnumbered by coastal sites without shells, with the presence or absence of shells at coastal sites being determined by whether or not large natural shell beds were immediately adjacent to a given location.

A more useful test of the intensification hypothesis would be the analysis of growth rates and size and mortality profiles of oyster shells and fish remains, or compound-specific isotope analysis of amino acids to examine a change in the ecosystem structure. Few such studies are available. Where they have been applied, they suggest changes in the Neolithic period rather than the Mesolithic^27,28^.

### Biomolecular evidence of palaeodiet

Lewis et al. cite Fischer et al.’s^29^ study in which stable isotope analysis of human bone collagen was undertaken as evidence for ‘a shift to a marine-based diet occurring at the boundary between the Maglemose and Kongemose culture’ (p. 5), with the onset of higher marine productivity. This interpretation does not take into account that Early Mesolithic coastlines throughout most of Southern Scandinavia are presently submerged and have received almost no investigation. Indeed, the sites in question were almost exclusively inland sites at the time, ‘miles away from the contemporaneous sea shores.’ (p. 2127) as pointed out by Fischer et al.^29^ themselves.

In a more recent study, stable isotope analysis of human bone collagen from Maglemosian sites on the uplifted west coast of Sweden has demonstrated that marine and freshwater foodstuffs^30^ contributed significantly to human diet, suggesting that a shift to a marine-based diet was not as significant a step-change as implied by Lewis et al.^1^.

### Fishing technology

Lewis et al. (their Fig. 2i) also cite evidence of the progressive increase in the range and variety of fishing equipment during the Late Mesolithic period as evidence for progressively intensified fishing practices. However, some technologies, such as fish hooks and water transport, already occurred in the Maglemosian period^31–34^, several millennia earlier than their figure indicates. Moreover, another crucial fishing technology, stationary fish weirs, which also date to as early as ca. 9000 cal BP, have been left out entirely, despite their importance in facilitating the mass capture of fish^25,26,35^. Even earlier than that, ca. 9200 cal BP, evidence for the conservation of large quantities of fish through fermentation has been identified at the site of Norje Sunnansund in Sweden^36^. We conclude that intensive fishing practices were already employed by the communities that colonised the earliest Littorina shorelines of the Danish Straits and that the available evidence of fishing techniques provides no support for progressive intensification of the fishing economy during the Mesolithic period on the scale proposed in Fig. 2i.

### Summary

We conclude that the methods, data, and assumptions used by Lewis et al. to support a hypothesis of Late Mesolithic population increase based on an intensified marine economy are not sufficiently substantiated. The Holocene environment of Southern Scandinavia was undoubtedly a highly dynamic one involving a complex web of changing interactions between climate, ecology, palaeogeography, and human societies. It would be surprising if there were not some interactions between these many variables, and palaeoecological data of the type produced by Lewis et al. have a role to play in such investigations. However, if we are to unravel the relationships between environmental changes and human responses, it will be necessary to develop collaborative research that better integrates palaeoecological, archaeological, marine geoscientific, and biomolecular data, pays more attention to the taphonomic history of archaeological sites and materials, and above all intensifies the investigation of submerged coastlines, where so much of the evidence required to discriminate between alternative hypotheses must be sought^37,38^.

## Data Availability

All data generated or analysed during this study are included in this published article.
